# Quantitative study on the fate of residual soil nitrate in winter wheat based on a ^15^N-labeling method

**DOI:** 10.1371/journal.pone.0171014

**Published:** 2017-02-07

**Authors:** Jing-Ting Zhang, Zhi-Min Wang, Shuang-Bo Liang, Ying-Hua Zhang, Shun-Li Zhou, Lai-Qing Lu, Run-Zheng Wang

**Affiliations:** 1 College of Agronomy & biotechnology, China Agricultural University/Key Laboratory of Farming System, Ministry of Agriculture, Beijing, China; 2 Institute of Cereal and Oil Crops, Hebei Academy of Agriculture and Forestry Sciences, Hebei, China; 3 Wuqiao Experimental Station, China Agricultural University, Hebei, China; University of Delhi, INDIA

## Abstract

A considerable amount of surplus nitrogen (N), which primarily takes the form of nitrate, accumulates in the soil profile after harvesting crops from an intensive production system in the North China Plain. The residual soil nitrate (RSN) is a key factor that is included in the N recommendation algorithm. Quantifying the utilization and losses of RSN is a fundamental necessity for optimizing crop N management, improving N use efficiency, and reducing the impact derived from farmland N losses on the environment. In this study, a ^15^N-labeling method was introduced to study the fate of the RSN quantitatively during the winter wheat growing season by ^15^N tracer technique combined with a soil column study. A soil column with a 2 m height was vertically divided into 10 20-cm layers, and the RSN in each layer was individually labeled with a ^15^N tracer before the wheat was sown. The results indicated that approximately 17.68% of the crop N derived from RSN was located in the 0–2 m soil profile prior to wheat sowing. The wheat recovery proportions of RSN at various layers ranged from 0.21% to 33.46%. The percentages that still remained in the soil profile after the wheat harvest ranged from 47.08% to 75.44%, and 19.46–32.64% of the RSN was unaccounted for. Upward and downward movements in the RSN were observed, and the maximum upward and downward distances were 40 cm and 100 cm, respectively. In general, the ^15^N-labeling method contributes to a deeper understanding of the fates of the RSN. Considering the low crop recovery of the RSN from deep soil layers, water and N saving practices should be adopted during crop production.

## Introduction

Nitrogen (N) fertilization is a common practice in agricultural production, and it plays a key role in achieving the desired crop yields because soils do not have sufficient N in available forms to support production levels. However, the N use efficiency (NUE) is low in many soils, usually at <50% globally [1]. The apparent NUE may be 30 to 35% for agricultural production in China [2], and it averaged only 33% in relation to cereal grain production [3]. Low N recovery by a crop is associated with N loss by leaching, volatilization, denitrification, and soil erosion. Air contamination by ammonia (NH_3_), nitrous oxide (N_2_O) and the other N oxides (NO and NO_x_) and groundwater contamination by nitrate have been recognized as major concerns for humanity. The application of mineral fertilizers in agriculture is the principal source of those contaminants [4]. There is a great deal of reported evidence to show that agricultural activities are associated with the nonpoint source pollution of groundwater by nitrate [5–8] and the emission of greenhouse gases related to N [9–12].

Plant N sources primarily include the mineralization of soil organic matter, residual inorganic N in soil, fertilization with synthetic N, and the biological fixation of atmospheric N_2_. There is a fundamental need for quantifying the utilization and losses of each N source under various defined circumstances so that the N management practices used in crop production can be optimized, the NUE can be improved and N losses can be decreased. The inorganic N in the soil, including ammonium-N (NH_4_^+^-N) and nitrate-N (NO_3_^−^-N), are not the only primary forms that plants can take up, but they are also involved in nitrate leaching loss processes and atmospheric loss by volatilization, nitrification and denitrification. The soil NH_4_^+^-N that is only present at the surface of the 20 cm-deep layer would exhibit a higher level within a short time following the application of N fertilizer. After that, it remained at a relatively low and constant level in the soil profile during the cropping season in the North China Plain (NCP) [13]. Therefore, the NH_4_^+^-N content did not reflect the available soil N level in this region [13–15]. Most fertilizers contain nitrate or forms of N (e.g., urea and ammonium) that can be converted into nitrate [16]. Urea that was applied to the soil was transformed into NO_3_^−^-N within 1 week in a winter wheat-summer maize rotation system [17]. Therefore, the considerable surplus N that accumulated in the soil profile would primarily be present in the form of NO_3_^−^-N after the harvest [18].

In the absence of N fertilizer, the residual inorganic N in the soil is lower. However, large amounts of inorganic N were accumulated in the soil profile when N fertilizer was applied, especially in the intensive crop production system. Fertilization has become an important measure for increasing the grain yield, because it could enhance the grain yield dramatically. In general, the N in Chinese agro-ecosystems has reached a surplus since the 1980s, and this N surplus amounted to an increase to 6.22 million tons in 1998 [19]. The NCP is one of the most intensively cultivated agricultural regions in the country, and it plays an irreplaceable role in Chinese food production. The winter wheat-summer maize rotation is the most important cropping system in the region, and it contributed more than 48% and 39% of the total wheat and maize production in China, respectively [20]. The annual application rate of N fertilizer for conventional agricultural practices ranged from 550–600 kg N ha^-1^ in the rotation system [21]. There was a considerable surplus of N [22] and high soil nitrate accumulation [23], and the surplus N reached 212 kg N ha^-1^ yr^-1^ under current farmer N application practices [24]. The average N balance of arable systems in Germany showed surplus N amounts of 110–130 kg N ha^-1^ yr^-1^, reaching 125–230 kg N ha^-1^ yr^-1^ in the intensive winter wheat-summer maize rotation of the Loess Plateau and 217–335 kg N ha^-1^ yr^-1^ in the rice-wheat double cropping system in the Taihu region of China [25]. The N surplus was 55–67 kg N ha^-1^ yr^-1^ in Europe according to the CAPRI agro-economic model [26].

Residual soil nitrate (RSN) is a double-edged sword. A soil test based on the RSN in the surface 30-cm layer has great promise for improving N management during corn production [27]. The rate of N fertilizer application was influenced by the RSN amount and the soil depth of the measurement when calculated by using the N requirement index [28]. In NCP, the winter wheat yield response to added N fertilizer was unlikely if the RSN exceeded 72 (0–30 cm soil depth) and 175 kg N ha^-1^ (0–90 cm soil depth) before the sowing and shooting stages, respectively [23]. The use of site-specific N management, in which the N recommendation algorithm for maize includes soil organic matter and RSN, could increase the NUE and reduce the environmental impact [29]. However, if the crops did not utilize the RSN, then downward leaching cannot be avoided because nitrate is soluble in water and is not retained by exchange processes in the soil. The large amounts of NO_3_^−^-N that are accumulated in the soil profile are readily leached down to deeper soil layers and would pollute the shallow groundwater eventually due to heavy rainfall or excessive irrigation [25]. Even with economic and optimum N fertilization in maize, 41–138 kg N ha^-1^ was still leached into the 1.2 m soil depth in the summer [30]. An average annual nitrate leaching loss of 29 kg N ha^-1^ was observed for non-fertilized, non-irrigated corn over a five-year period in central Minnesota [31]. In Britain, the amount of NO_3_^−^-N leaching loss increased by 36 kg N ha^-1^ yr^-1^ on an intensively cultivated wheat field over a 50-yr period [32]. In southeastern Minnesota, approximately 15% of the applied N, 68% of the RSN in the non-root zone layer, and 20% of the RSN in the root zone layer were deposited into the groundwater annually in a maize field [33]. N fertilization improved the soil nitrate content and enhanced the nitrate leaching loss [34–35]. Drinking water with an NO_3_^−^-N content higher than 11.3 mg NO_3_^−^-N L^-1^ (50 mg nitrate L^-1^) is considered unsafe for human consumption [36]. An investigation of ground and drinking water NO_3_^−^-N concentrations that was conducted in 14 cities and counties in northern China showed that the concentrations at over half of the 69 locations exceeded this limit, with the highest nitrate concentration reaching 300 mg L^-1^ [37].

Isotopic tracer and isotope discrimination techniques are now being used widely in qualitative and quantitative research on N cycling in organisms and ecosystems. The nitrate sources in rivers can be determined using ^15^N, ^17^O, and ^18^O by mixing models according to the distinct isotopic characteristics of nitrate from different sources [38]. However, because indigenous soil nitrate has no distinct isotopic characteristics for nitrate that is related to a special soil type, it is difficult to discriminate the RSN from other N and assess its fate in a quantitative sense by using the isotope discrimination technique. Through the exogenous ^15^N injection (^15^N tracer) technique, a certain amount of exogenous nitrate that is labeled by ^15^N is injected into a soil layer to form a particular accumulation state of ^15^N-labeled nitrate, which is commonly adopted to study the fate of accumulated nitrate in the soil profile [39–42]. However, the absolute content of NO_3_^−^-N in the labeled soil layer will dramatically increase after exogenous ^15^NO_3_^−^-N injection and will not coordinate with the status quo ante, although the crop recovery and migration distance of injected exogenous ^15^N can be defined using this technique. Moreover, the injected ^15^NO_3_^−^-N primarily concentrates in the small amount of soil surrounding the injection point, and it leads to a dramatic increase in the NO_3_^−^-N concentration at the injection point. The non-uniformity of exogenous ^15^NO_3_^−^-N distribution in the soil and the dramatic increase of the NO_3_^−^-N concentration in the injection point will have a strong influence on the results and lead to a large deviation in the real situation, so the exogenous ^15^N injection technique is not expected to be the best in terms of quantitative research on N cycling.

We qualitatively and quantitatively studied the fate of N fertilizer using a ^15^N tracer technique in a winter wheat-summer maize rotation system in NCP [42–43]. In this paper, we introduced a novel method for labeling the indigenous soil NO_3_^−^-N in situ, through which the inherent accumulation state of RSN would not be changed. The feasibility of this method was evaluated and the fates of the RSN in different soil layers before winter wheat sowing were determined.

## Materials and methods

### Experimental site description

This study was conducted at Wuqiao Experimental Station of China Agricultural University in Wuqiao County of Hebei Province, China. Wuqiao County (37°29′–37°47′ N; 116°19′–116°42′ E) is located in the middle of the NCP. The area has a warm-temperate, sub-humid continental monsoon climate, with cold winters and hot summers. The annual cumulative mean temperature for days above 10°C is 4000 to 5000°C, and the annual frost-free period is 175 to 220 d. The altitude is 14–22.6 m above sea level, and the average groundwater table is 6–9 m. The average annual rainfall for the last 33 yrs has been 542 mm, with a sharp yearly fluctuation and erratic seasonal distribution. The average rainfall during the winter wheat growth period is 117 mm, accounting for 21.6% of the total annual rainfall. Our studies did not involve endangered or protected species, and no specific permissions were required since the experimental activities were carried out at the Wuqiao Experimental Station, which is a regional research station of China Agricultural University.

The soil at the site is classified as a Calcaric Fluvisol with a sandy clay loam texture. The soil pH, organic matter content, total N, NO_3_^−^-N and NH_4_^+^-N concentration, Olsen-P, NH_4_OAc-extractable K and bulk density from the 0 to 2 m soil profile depth at 0.2 m intervals are presented in Table 1.

**Table 1:** Selected physical and chemical properties for the experimental soil.

Soil layer (cm)	pH (H_2_O)	Organic matter (%)	Total N (g kg^-1^)	NO_3_^−^-N (mg kg^-1^)	NH_4_^+^-N (mg kg^-1^)	Olsen-P (mg kg^-1^)	NH_4_OAc-K (mg kg^-1^)	Bulk density (g cm^-3^)
0–20	8.53	1.13	1.00	14.53	1.54	24.8	188.8	1.34
20–40	8.64	0.56	0.46	11.34	1.23	4.98	91.55	1.40
40–60	8.63	0.39	0.34	22.16	1.19	3.45	61.60	1.46
60–80	8.54	0.38	0.27	31.07	1.67	2.64	67.51	1.47
80–100	8.51	0.34	0.24	17.52	0.91	3.31	66.39	1.48
100–120	8.59	0.30	0.24	14.14	1.32	3.59	66.55	1.45
120–140	8.69	0.25	0.18	9.65	1.26	3.40	52.86	1.48
140–160	8.77	0.19	0.15	9.72	1.56	3.03	36.64	1.44
160–180	8.76	0.19	0.14	11.62	1.95	1.60	37.02	1.50
180–200	8.75	0.17	0.14	13.56	1.18	2.95	38.94	1.48

### RSN labeling in situ

**Fig 1:**
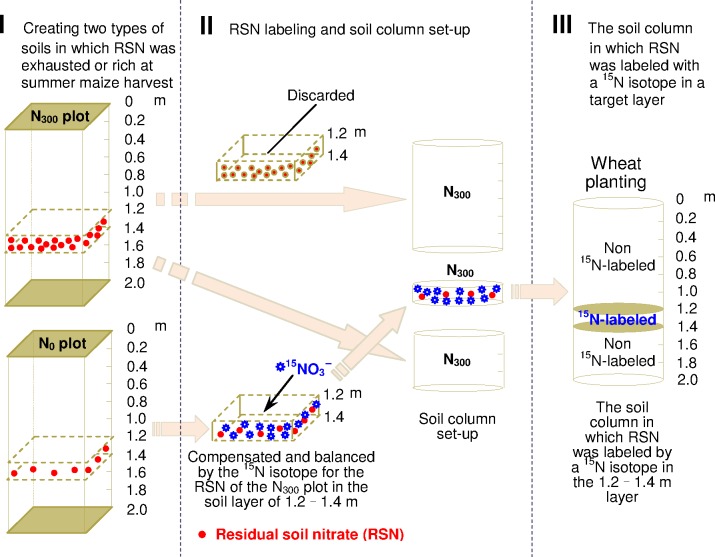
Sketch showing the process for residual soil nitrate (RSN) labeling in situ and the soil column setup in which a selected soil layer was labeled with the ^15^N isotope (the layer from 1.2 to 1.4 m was used as an example). N_0_, no N fertilizer applied to each crop in the winter wheat-summer maize rotation system; N_300_, 300 kg N ha^-1^ applied to each crop in the rotation system.

To label RSN in situ and quantify the fates of RSN in different soil layers before winter wheat sowing, a soil column study was conducted using a ^15^N tracer technique. The RSN labeling process and the soil column setup in which a selected soil layer was labeled with ^15^NO_3_^−^-N was employed as follows (Fig 1):

First, a typical farmer's field was selected and two types of representative soils were created in which the RSN was exhausted or rich at the summer maize harvest. Prior to the experiment, a typical field that was uniform in soil fertility and under continuous winter wheat-summer maize rotation using traditional tillage for 2 years was selected as the target field. The field was divided into two parts, in which one received no N fertilizer during the winter wheat and summer maize growing period (N_0_ plot) and the other received 300 kg N ha^-1^for each crop according to the annual application rate for N fertilizer used in conventional agricultural practices for the winter wheat-summer maize rotation system (N_300_ plot) [21]. Plot size was 5 m×5 m, and a 2 m wide isolation belt was set up between the two plots to avoid possible interference. The two plots were treated over a winter wheat and summer maize rotation season, and the same typical farmer's practices were implemented in the two plots, except for the N fertilization practice. The N fertilizer in the N_300_ plot was applied in two splits for each crop, with 150 kg N ha^-1^ (urea) being broadcast before tillage and the remaining 150 kg N ha^-1^ (urea) broadcast followed by irrigation at the jointing stage for winter wheat; a 120 kg N ha^-1^ rate (urea) was applied at sowing and the remaining 180 kg N ha^-1^ (urea) was top-dressed at the thirteen-leaf stage for summer maize. At the first application of N fertilizer for each crop, 103.5 kg P_2_O_5_ ha^-1^, 162.7 kg K_2_O ha^-1^, and 30 kg ZnSO_4_ ha^-1^ were also applied to the two plots according to local recommendations. These fertilizers were applied into 3–5 cm-deep furrows and covered with the summer maize soil. For winter wheat planting, 750 m^3^ ha^-1^ irrigation was applied, the first fertilization was performed, and then the field was plowed and leveled. The wheat cultivar Shijiazhuang 8 was sown on October 18, 2007 and harvested on June 13, 2008. After the winter wheat harvest, the hybrid maize cultivar Zhengdan 958 was sown on June 13, 2008, with zero tillage. After a rotation cycle, a difference in the RSN content of the soil profile between the two plots should occur because of the plant uptake and the nitrate movement during the winter wheat and summer maize growing period. It is obvious that the RSN content for a soil layer in the N_300_ plot should be higher than that for the corresponding layer in the N_0_ plot.

Second, the RSN was labeled in situ. After the summer maize harvest, the RSN content for each soil layer of the two plots was determined. Soil samples were taken from the 0 to 2 m profile depth at 0.2 m intervals from four sites per plot using a soil auger with a 4 cm diameter. These soil samples were extracted with a 1:10 ratio of soil:0.01 mol L^-1^ CaCl_2_ and analyzed for NO_3_^−^-N using Continuous Flow Analysis (TRAACS 2000). According to the results for the RSN content analysis at each soil layer from the 0 to 2 m profile depth, the difference in the NO_3_^−^-N content of each soil layer between the two plots was calculated. Compared to the N_300_ plot, the NO_3_^−^-N deficit in the N_0_ plot for the soil layer was compensated by exogenous ^15^NO_3_^−^-N, and it showed that the NO_3_^−^-N content for the layer in the N_0_ plot was equal to that for the corresponding layer in the N_300_ plot. After this point, the RSN content for the soil layer between the two plots was the same, and the soil that was compensated with exogenous ^15^NO_3_^−^-N for a soil layer in the N_0_ plot could be used to represent the RSN status of the corresponding layer in the N_300_ plot. The RSN for the soil layer in the N_300_ plot was labeled using a ^15^N isotope.

Finally, the ^15^N amount was calculated and the soil column was set up. According to the description above, the theoretical compensated amount of ^15^N was calculated using the following equation:

$${\text{m}}_{15\text{N}}=\text{M}\times \left({\text{c}}_{1}-{\text{c}}_{2}\right)$$

where the m_15N_ was the theoretical amount of ^15^N that was incorporated into the soil to compensate for the NO_3_^−^-N deficit between the two plots (mg), the dry weight of the soil to be labeled was described as M (kg), and c_1_ and c_2_ (mg kg^-1^ DW) represented the NO_3_^−^-N content for a soil layer in the N_300_ and N_0_ plots, respectively.

To quantify the fate of the RSN at the summer maize harvest during the winter wheat growing period, a soil column study was conducted by creating soil columns in which a selected soil layer was labeled with ^15^N isotope before sowing the wheat (Fig 1). In this study, only part of the soil for each soil layer in the N_300_ plot was substituted by the soil of the corresponding layer in the N_0_ plot. For the residual soil NO_3_^−^-N content in each soil layer from the two plots after summer maize harvest, the total soil dry weight of each layer for a column and the soil amount to be labeled by ^15^N isotopes in each layer are presented in S1 Table. At that point, the amount of ^15^N isotope that was incorporated into each soil layer was calculated by the m_15N_ multiplied by the ^15^N atom abundance of the ^15^N-labeled chemical. The ^15^N-labeled KNO_3_ (99.21% ^15^N atom abundance) was used in this study, the incorporated amount of ^15^N isotope and the ratios of ^15^N to total NO_3_^−^-N for each soil layer are also given in S1 Table. The amount of K^15^NO_3_ that was incorporated into each soil layer was calculated on the basis of the incorporated amount of ^15^N isotope for each soil layer and the mass fraction of ^15^N in K^15^NO_3_.

Fig 1 shows a sketch of the RSN labeling in situ and the soil column setup in which the 1.2 to 1.4 m layer was used as an example of ^15^N isotope labeling. The soil around the sampling site for nitrate content determination was taken from the two plots at 0.2 m intervals to a 2 m depth, which were mixed and stacked separately to set up the soil columns. All the soil for the column setup was taken from the N_300_ plot except the labeled soil layer, and only one layer was labeled with ^15^N isotope in a column. Part of the soil for the labeled soil layer was taken from the N_0_ plot, and the K^15^NO_3_ (99.21% ^15^N atom abundance) was incorporated and mixed evenly before it was used for the column set-up. Two hollow iron column molds measuring 1 m in length and 36 cm in diameter were used to set up the soil column to a 2 m height, and the setup method was derived from Wu et al. [43]. In this experiment, 44 columns were made in all, with 40 for the RSN labeling of the ten soil layers and the other 4 columns as a control (all of the soil for the column setup was taken from the N_300_ plot). To alleviate or avoid the influence from the adjacent ^15^N-labeled soil columns resulting from ^15^NO_3_^−^-N leaching below the 2 m soil depth, these soil columns were arranged according to the rule in which the distance for the ^15^N labeled soil layer between the adjacent soil columns should be the greatest.

### Experimental management

The winter wheat cultivar Shijiazhuang 8 was sown at 60 seeds per column on October 18, 2008. At the third leaf stage (On November 12, 2008), 55 healthy and vigorous seedlings were retained per column based on local recommendations for the plant density. To apply fertilizer to each column, 10 cm of the topsoil was removed from the column and mixed with 1.575 g N, 1.385 g P_2_O_5_, 1.130 g K_2_O, and 0.3 g ZnSO_4_ according to the recommended fertilizer rate for water-saving winter wheat [44]. The column was then refilled with this mixture before sowing. For each soil column, 75 mm of water was added by irrigation prior to fertilization, and 75 mm was added at the jointing and flowering stages, for a total application of 225 mm during the winter wheat growth period. The rainfall was 148.5 mm during the wheat growth period. The wheat was harvested on June 6, 2009.

### Sampling and laboratory analyses

The aboveground plants from each column were harvested at wheat physiological maturity. The straw and grain were separated, oven-dried at 105°C for 30 min and then decreased to 80°C until they reached a constant weight. The dried plant samples were weighed, milled, and screened through a 2 mm sieve to analyze the total N and ^15^N enrichment. After the harvest, soil samples were collected from the middle of each column in 0.2 m increments, to a depth of 3 m. Some soil samples were air-dried, milled, and screened through a 0.9 mm sieve to detect the ^15^N enrichment. The ^15^N enrichment of soil and plant samples was analyzed using a ZHT-03 mass spectrometer (Beijing Analysis Instrument Co. Ltd., Beijing China).

### Rate calculation

The ^15^N uptake and RSN recovery amounts from each labeled soil layer by wheat and residual ^15^N amount in the soil were calculated as follows:

$${}^{15}\text{N}\;\text{uptake amount in the wheat grains at harvest}\;(\text{mg})=\text{grain dry weight}\;(\text{g})\times \text{N concentration in grain}\;(\%)\times [{}^{15}\text{N}\;{\text{enrichment}\;(\%)}_{\text{sample}}-{}^{15}\text{N}\;{\text{enrichment}\;(\%)}_{\text{background}}]÷10$$

$${}^{15}\text{N}\;\text{uptake amount in the wheat straw at harvest}\;(\text{mg})=\text{straw dry weight}\;(\text{g})\times \text{N concentration in straw}\;(\%)\times [{}^{15}\text{N}\;{\text{enrichment}\;(\%)}_{\text{sample}}-{}^{15}\text{N}\;{\text{enrichment}\;(\%)}_{\text{background}}]÷10$$

$$\text{Total}\;{}^{15}\text{N}\;\text{uptake by wheat at harvest}\;(\text{mg})={}^{15}\text{N}\;\text{uptake amount in the grain}\;(\text{mg})+{}^{15}\text{N}\;\text{uptake amount in the straw}\;(\text{mg})$$

$$\text{RSN recovery from each labeled soil layer by wheat}\;(\text{mg})=\text{Total}\;{}^{15}\text{N}\;\text{uptake by wheat at harvest}\;(\text{mg})÷[{}^{15}\text{N}\;\text{amount incorporated in the soil layer}\;(\text{mg})÷\text{total RSN amount in the soil layer before wheat sowing}\;(\text{mg})]$$

$$\text{Residual}\;{}^{15}\text{N}\;\text{in the soil layer at wheat harvest}\;(\text{mg})=\text{soil dry weight of the layer}\;(\text{kg})\times \text{soil N content}\;(\%)\times [{}^{15}\text{N}\;{\text{enrichment}\;(\%)}_{\text{sample}}-{}^{15}\text{N}\;{\text{enrichment}\;(\%)}_{\text{background}}]\times 100$$

### Statistical analysis

The primary data were processed using Microsoft Excel. The differences among the treatments were determined using an analysis of variance (ANOVA). The means were compared by finding the least significant differences (LSD) at the 0.05 level of probability. Statistical analyses were performed using the DUNCAN procedures in the SAS software package (SAS Institute, 1996).

## Results

### Wheat growth and N uptake

Approximately half of the soil from the labeled layer should have been used from the N_300_ plot, but it was replaced by the soil from the N_0_ plot to label the RSN by ^15^N tracer (S1 Table). This replacement did not affect the wheat growth. Both the aboveground biomass and N uptake of winter wheat grew in the soil columns in which one layer was labeled, and there was no significant difference compared to the control column (CK) in which all of the soil came from the N_300_ plot (Table 2).

**Table 2:** Dry matter accumulation and N uptake of winter wheat for each labeled soil layer under the soil column conditions. Means followed by the same letter within the same column are not significantly different at P < 0.05.

Soil layer labeled by ^15^N (cm)	Straw (g column^-1^)		Grain (g column^-1^)		Straw + grain (g column^-1^)	
	Dry matter	N uptake	Dry matter	N uptake	Dry matter	N uptake
0–20	55.27 a	0.27 a	59.22 a	1.32 a	114.5 a	1.59 a
20–40	56.52 a	0.26 a	60.52 a	1.36 a	117.0 a	1.62 a
40–60	53.39 a	0.26 a	61.53 a	1.36 a	114.9 a	1.62 a
60–80	55.52 a	0.26 a	60.78 a	1.35 a	116.3 a	1.61 a
80–100	55.76 a	0.26 a	61.58 a	1.39 a	117.3 a	1.66 a
100–120	53.72 a	0.27 a	57.34 a	1.28 a	111.1 a	1.55 a
120–140	57.73 a	0.29 a	61.92 a	1.39 a	119.6 a	1.68 a
140–160	54.58 a	0.28 a	59.65 a	1.39 a	114.2 a	1.67 a
160–180	54.42 a	0.25 a	58.83 a	1.31 a	106.2 a	1.57 a
180–200	53.11 a	0.27 a	57.55 a	1.38 a	107.7 a	1.65 a
CK	55.51 a	0.27 a	58.74 a	1.33 a	114.2 a	1.60 a

### The fate of the RSN

The three outlets for the fate of RSN consisted of crop recovery, RSN remaining in the soil profile and loss by leaching or denitrification. Whether the NO_3_^−^-N is located at different soil layers could be absorbed by crops depends on the developmental stage of the crop and the depth to which the crop roots could reach. Winter wheat could utilize the soil NO_3_^−^-N that was distributed in the soil profile to 2 m deep in this study. A negative relationship was found between the recovery rate of ^15^NO_3_^−^-N at different soil layers by wheat and the soil depth of the ^15^NO_3_^−^-N was identified. The deeper the soil layer, the lower the recovery rate by the wheat. Approximately 0.21% to 33.46% of ^15^NO_3_^−^-N for the ten labeled soil layers was absorbed by wheat (Table 3). This finding may be explained by the distribution pattern of winter wheat roots in the soil profile. A study that was conducted in this region showed that the winter wheat roots were able to reach a 2 m soil depth. The root distribution in soil was 71.1% for 0–0.6 m, 25.0% for 0.6–1.2 m, and 3.9% for the 1.2–2.0 m depth [42].

**Table 3:** The fate of ^15^NO_3_^−^-N that was incorporated into each soil layer after a winter wheat growing season under the soil column conditions.

Soil layer labeled by ^15^N (cm)	Incorporated ^15^N amount (mg)	Crop recovery				Residue in soil		Unaccounted for ^15^N	
		Straw (mg column^-1^)	Grain (mg column^-1^)	Total (mg column^-1^)	Percentage of crop recovery to incorporated ^15^N (%)	Amount (mg column^-1^)	Percentage of residue in soil to incorporated ^15^N (%)	Amount (mg column^-1^)	Percentage of unaccounted for ^15^N to incorporated ^15^N (%)
0–20	145.62	6.58	42.14	48.72	33.46	68.56	47.08	28.34	19.46
20–40	151.19	3.93	32.51	36.44	24.10	82.11	54.31	32.64	21.59
40–60	163.05	1.86	18.95	20.82	12.77	105.57	64.75	36.65	22.48
60–80	184.30	0.75	6.87	7.61	4.13	139.04	75.44	37.65	20.43
80–100	211.56	0.57	3.92	4.49	2.12	158.67	75.00	48.40	22.88
100–120	189.35	0.39	2.05	2.45	1.29	142.07	75.03	44.84	23.67
120–140	112.83	0.16	1.11	1.27	1.13	80.58	71.42	30.97	27.45
140–160	118.03	0.12	0.67	0.78	0.66	87.84	74.42	29.41	24.92
160–180	149.17	0.06	0.30	0.36	0.24	103.99	69.71	44.83	30.05
180–200	160.55	0.06	0.28	0.34	0.21	107.81	67.15	52.40	32.64

Approximately 47.08% to 75.44% of the ^15^NO_3_^−^-N that was incorporated into the labeled soil layers still remained in the soil profile after wheat harvest, and the residual rates of ^15^NO_3_^−^-N in the shallow labeled soil layers were lower than that in the deeper layers (Table 3).

A considerable amount of ^15^NO_3_^−^-N in each labeled soil layer was unaccounted for after the winter wheat harvest, and it ranged from 19.46–32.64% (Table 3).

### The movement and distribution pattern of the ^15^N remaining in the soil profile

**Fig 2:**
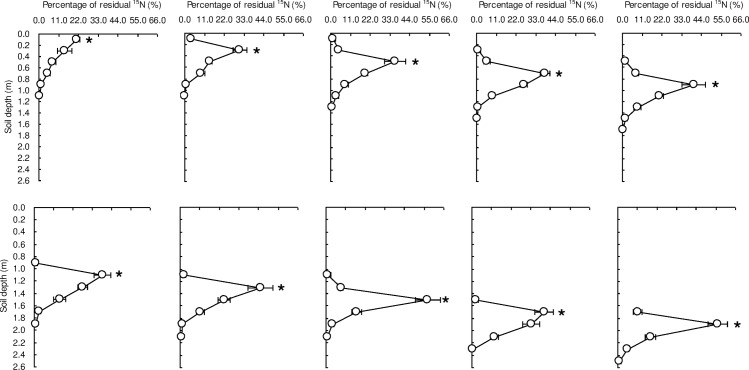
Distribution pattern of residual ^15^N in the soil profile at winter wheat harvest. * denotes that the soil layer was labeled with K^15^NO_3_ (99.21% ^15^N atom abundance) before winter wheat sowing.

The chemical characteristics of NO_3_^−^ make this molecule susceptible to migrating downward or upward with the flow of soil water. The vertical movement of labeled NO_3_^−^-N in the soil profile occurred during the winter wheat growing season for all the layers. The deeper the placement of ^15^N-labeled nitrate, the shorter the distance of the downward movement. The maximum upward migrating distance was 40 cm and the maximum downward migrating distance was 100 cm during wheat growing season (Fig 2). The upward or downward movement of NO_3_^−^-N is closely related to the soil water movement. At some growth stages in water-saving wheat, a soil water deficit occurs in the upper soil layer because of crop water consumption and soil evaporation. In this case, the soil NO_3_^−^-N in the deeper layer might move upwards in concert with the soil moisture movement. The distance of the NO_3_^−^-N downward movement in the topsoil was greater than that in the subsoil. This finding may be explained by the soil water movement, which was derived from irrigation or rainfall during the crop growing season.

After the winter wheat harvest, the highest percentage of residual ^15^NO_3_^−^-N in a soil layer to the total amount of residual ^15^NO_3_^−^-N in the soil column was observed at the ^15^N-labeled layer for all ten ^15^N-labeled layers, and it ranged from 21.23–57.06%. These percentages declined with the increasing distance from the ^15^N-labeled layer in the soil profile for all ten ^15^N-labeled layers, and they showed a single peak curve, except the ^15^N-labeled layer for 0–0.2 m (Fig 2).

### The contribution of RSN to winter wheat N nutrition

The total N uptake of winter wheat over its whole life under the given soil column conditions ranged from 1.55 g column^-1^ to 1.68 g column^-1^ for the ten ^15^N-labeled soil layers, and the RSN absorbed by the crop from the ^15^N-labeled layer ranged from 0.82 mg column^-1^ to 127.54 mg column^-1^. Thus, the RSN contribution at summer maize harvest to winter wheat N nutrition ranged from 0.05% to 8.02%, and it showed a sharp decrease with the increasing soil depth. In general, the contribution that was derived from the RSN accumulated in the 0–2 m soil profile before sowing for winter wheat N nutrition was 17.68% (Table 4).

**Table 4:** The contribution of residual soil nitrate (RSN) at summer maize harvest to winter wheat N nutrition under the soil column conditions.

Soil layer labeled by ^15^N (cm)	Total N uptake amount (g column^-1^)	^15^N uptake amount (mg column^-1^)	Percentage of incorporated ^15^N to RSN of N_300_ plot at summer maize harvest (%)	RSN uptake amount from the labeled layer (mg column^-1^)	Contribution (%)
0–20	1.59	48.72	38.20	127.54	8.02
20–40	1.62	36.44	48.64	74.91	4.63
40–60	1.62	20.82	46.91	44.37	2.74
60–80	1.61	7.61	42.50	17.91	1.11
80–100	1.66	4.49	43.40	10.34	0.62
100–120	1.55	2.45	48.13	5.08	0.33
120–140	1.68	1.27	41.17	3.09	0.18
140–160	1.67	0.78	43.07	1.82	0.11
160–180	1.57	0.36	43.71	0.82	0.05
180–200	1.65	0.34	40.86	0.84	0.05
	1.62†			286.72‡	17.68

†, Average value of the ten ^15^N-labeled soil layers for the total N uptake by winter wheat.

‡, Total for the ten ^15^N-labeled soil layers in the residual soil nitrate (RSN) uptake of winter wheat.

## Discussion and conclusions

### The feasibility and validity of the ^15^N-labeling method for quantitatively monitoring RSN

Plant N sources primarily include the mineralization of soil organic matter, the residual inorganic N in soil, N fertilization, and the biological fixation of atmospheric N_2_. It is fundamentally necessary to quantify the utilization and losses of each N source under various defined circumstances. However, there has been no reasonable method for labeling and quantitatively monitoring indigenous soil NO_3_^−^-N until the present. In this study, we introduced a method for labeling the RSN that was located in a certain position within the soil profile by employing a ^15^N tracer technique combined with a soil column study. The ^15^N-labeling of RSN for farmland-implemented conventional agricultural practices was achieved by incorporating an equal amount of exogenous ^15^NO_3_^−^-N into a nitrate-exhausted soil and then using the ^15^N-labeled, exhausted soil to replace part of the target field soil of the corresponding layer. The nitrate-exhausted soil was created by crop absorption under no N fertilizer application. However, the amounts of nutrient elements (N, P, and K) removed by crops were much smaller in the N_0_ plot than in the N_300_ plot because the crop growth was limited by N stress in the N_0_ plot (S2 Table). Theoretically, the differences in nutrient uptake should have different influences on the soil chemical properties for the two plots. However, there were no significant differences in the primary soil chemical properties in the 0–2 m soil profile between the two plots after a wheat-maize rotation (S3 Table). This finding is likely to be explained by the good buffering role that soil plays in maintaining the stability of the soil chemical properties that were unaffected by the agricultural practices. Moreover, in this experiment, only approximately half of the soil was taken from the N_0_ plot for the ten layers of labeling, so the effects of the differences in NH_4_^+^-N, microelements and others between the two plots on the wheat growth should also be disregarded. As a result, the wheat growth was not influenced after part of the soil for a layer that should come from the N_300_ plot was replaced by the soil of the corresponding layer from the N_0_ plot (Table 2).

To quantify the fate of the RSN, a soil column study was conducted. However, the growth of the winter wheat that was raised in the soil column was restricted compared with the grown in the field under the same management, and the grain yield and aboveground biomass per square meter decreased by 15.6% and 14.7%, respectively. The likely reason was that the soil bulk density in each layer might not be the same as that in the field, and it is inclined to go higher because it is difficult to monitor the soil moisture content during the soil column setup procedure [43]. Nevertheless, it was possible to build appropriate soil columns and get the plants to grow normally [43]. In any case, the ^15^N-labeling method is feasible and valid for quantitatively monitoring RSN, although it involves a complicated procedure.

### Crop recovery, migrating and loss of RSN

Our results indicated that the RSN was an important N nutrition source for wheat growth because the contribution derived from the RSN that accumulated in the 0–2 m soil profile for winter wheat N nutrition was 17.68%. However, the highest recovery percentage for ^15^NO_3_^−^-N was 33.46% in the 0–20 cm soil layer, and it decreased sharply with the increasing soil depth, reaching no more than 5% in the 60–200 cm layers (Table 3). The lower crop recovery of RSN leads to a higher leaching risk to the subsoil and eventually to groundwater. Therefore, viewing the RSN as a crop N nutrition source is inadvisable, and the fundamental strategy is to decrease the nitrate accumulation in the soil through optimal N management practices.

The vertical movement of the RSN in the soil profile was observed during the winter wheat growing season for all the ^15^N-labeled soil layers. The migration not only occurred downward but also occurred upward. The maximum migrating distance was 40 cm upward and 100 cm downward during the wheat growing season (Fig 2). The downward movement of RSN leads to a higher risk of leaching, but upward movement increases its availability to crops. Thus, from this perspective, deficit irrigation or water-saving management is an effective practice for decreasing soil nitrate leaching losses and improving its availability to crops.

At wheat harvest, 19.46–32.64% of the ^15^NO_3_^−^-N that was incorporated into the labeled soil layers was unaccounted for, and the percentage in the deep soil layers was higher (Table 3). In theory, there might be two outlets for the unaccounted for ^15^N in this study, with one being the ^15^N that remained in the wheat roots, and the other from gaseous loss via denitrification. However, denitrifying bacteria are heterotrophic microbes, and the soil organic matter content of the deep soil layers (>40 cm) was very low (Table 1), so these conditions might not be conducive to denitrification. Moreover, the root distribution in the deep subsoil was quite small, so the amount of ^15^N remaining in the roots should be correspondingly rare. Therefore, this finding might be related to large ^15^N enrichment measurement errors in the deep soil layer where the total N content was quite low, which would lead to a result in which the ^15^N enrichment was lower than the actual value.

## Supporting information

S1 Table The incorporated amount of ^15^N isotope in each labeled soil layer. (PDF)

S2 Table Effects of two N fertilizer rates on the N, P, and K uptake in winter wheat and summer maize. (PDF)

S3 Table Effects of two N fertilizer rates on the selected chemical properties of the soil after a winter wheat-summer maize rotation cycle. Means followed by the same letter within the same row for the same parameter are not significantly different at P<0.05.
(PDF)
