# Supplementary material for: Quantitative study on the fate of residual soil nitrate in winter wheat based on a 15N-labeling method
Source: PLoS One. 2017 Feb 7;12(2):e0171014. doi: 10.1371/journal.pone.0171014 (PMC5295662; doi:10.1371/journal.pone.0171014)
Supplement: S1 Table — (PDF) [file pone.0171014.s001.pdf]

Supporting Information

S1 Table. The incorporated amount of ^{15}N isotope in each labeled soil layer.

Soil layer labeled by ^{15}N (cm)	Total soil dry weight of each layer for a column (kg)	Soil dry weight to be labeled by ^{15}N in each layer (kg)	Residual soil NO_3^--N content in N_{300} plot § (mg kg^{-1})	Residual soil NO_3^--N content in N_0 plot § (mg kg^{-1})	Incorporated amount of ^{15}N isotope (mg)	^{15}N / total NO_3^--N (%)
0–20	26.23	13.12	14.53	3.34	145.62	38.20
20–40	27.41	13.70	11.34	0.22	151.19	48.64
40–60	28.58	14.29	12.16	0.66	163.05	46.91
60–80	28.78	14.39	15.07	2.16	184.30	42.50
80–100	27.82	13.91	17.52	2.19	211.56	43.40
100–120	27.82	13.91	14.14	0.42	189.35	48.13
120–140	28.40	14.20	9.65	1.64	112.83	41.17
140–160	28.19	14.10	9.72	1.28	118.03	43.07
160–180	29.37	14.68	11.62	1.38	149.17	43.71
180–200	28.97	14.49	13.56	2.39	160.55	40.86

§ , The residual soil NO_3^--N content for each soil layer was determined at summer maize harvest stage.
