# Supplementary material for: Quantitative study on the fate of residual soil nitrate in winter wheat based on a 15N-labeling method
Source: PLoS One. 2017 Feb 7;12(2):e0171014. doi: 10.1371/journal.pone.0171014 (PMC5295662; doi:10.1371/journal.pone.0171014)
Supplement: S2 Table — (PDF) [file pone.0171014.s002.pdf]

Supporting Information

S2 Table. Effects of two N fertilizer rates on the N, P, and K uptake in winter wheat and summer maize.

Treatment	N uptake amount (kg ha^{-1})			P uptake amount (kg ha^{-1})			K uptake amount (kg ha^{-1})		
	Winter wheat	Summer maize	Total	Winter wheat	Summer maize	Total	Winter wheat	Summer maize	Total
N_0	158.3	70.1	228.4	26.0	24.1	50.1	153.5	123.9	277.4
N_{300}	225.7	221.5	447.2	48.9	28.9	77.8	294.6	201.0	495.6
Significant	*	*	*	*	*	*	*	*	*

*, Significant difference at 0.05 level of probability within the same column.
