# Supplementary material for: Quantitative study on the fate of residual soil nitrate in winter wheat based on a 15N-labeling method
Source: PLoS One. 2017 Feb 7;12(2):e0171014. doi: 10.1371/journal.pone.0171014 (PMC5295662; doi:10.1371/journal.pone.0171014)
Supplement: S3 Table — Means followed by the same letter within the same row for the same parameter are not significantly different at P<0.05. (PDF) [file pone.0171014.s003.pdf]

Supporting Information

S3 Table. Effects of two N fertilizer rates on the selected chemical properties of the soil after a winter wheat-summer maize rotation cycle. Means followed by the same letter within the same row for the same parameter are not significantly different at P<0.05.

Soil layer (cm)	Total N (g kg^{-1})			Olsen-P (mg kg^{-1}) §			NH_4OAc-K (mg kg^{-1})			Organic matter (%)			pH (H_2O)		
	Before wheat sowing	After the cycle		Before wheat sowing	After the cycle		Before wheat sowing	After the cycle		Before wheat sowing	After the cycle		Before wheat sowing	After the cycle	
		N_0	N_{300}		N_0	N_{300}		N_0	N_{300}		N_0	N_{300}		N_0	N_{300}
0–20	0.96a	1.05a	1.00a	24.9a	35.3a	24.8a	126.2b	196.5a	188.8a	1.06a	1.09a	1.13a	8.37a	8.59a	8.53a
20–40	0.47a	0.38a	0.46a	2.81b	4.28ab	4.98a	122.83a	96.90a	91.55a	0.67a	0.62a	0.56a	8.28b	8.64a	8.64a
40–60	0.34a	0.29a	0.34a	1.37a	2.46a	3.45a	96.53a	96.76a	61.60b	0.40a	0.38a	0.39a	8.25b	8.54a	8.63a
60–80	0.23a	0.25a	0.27a	0.29b	1.88a	2.64a	60.0a	79.84a	67.51a	0.21a	0.34a	0.38a	8.24b	8.53ab	8.54a
80–100	0.16a	0.21a	0.24a	0.42b	2.18ab	3.31a	54.82a	68.10a	66.39a	0.16a	0.26a	0.34a	8.33a	8.42a	8.51a
100–120	0.18a	0.22a	0.24a	0.59b	1.76ab	3.59a	57.24a	73.14a	66.55a	0.27a	0.32a	0.30a	8.56a	8.60a	8.59a
120–140	0.18a	0.17a	0.18a	0.48a	2.03a	3.40a	52.38a	60.72a	52.86a	0.19a	0.20a	0.25a	8.62a	8.70a	8.69a
140–160	0.13a	0.14a	0.15a	0.41a	2.60a	3.03a	38.11ab	44.37a	36.64b	0.17b	0.18a	0.19a	8.63a	8.72a	8.77a
160–180	0.15a	0.13a	0.14a	0.58a	1.88a	1.60a	48.38a	46.01a	37.02a	0.08b	0.18ab	0.19a	8.55a	8.74a	8.76a
180–200	0.13a	0.13a	0.14a	0.52b	1.37b	2.95a	41.82a	46.11a	38.94a	0.06b	0.19a	0.17a	8.46a	8.75a	8.75a

§, Maybe some errors occurred during Olsen-P determination because the data before wheat sowing in subsoil layers were obviously lower than that after the cycle and there was no significant differences in statistics (the variation among replications were violent), too.
